# Classification of IDH wild-type glioblastoma tumorspheres into low- and high-invasion groups based on their transcriptional program

**DOI:** 10.1038/s41416-023-02391-y

**Published:** 2023-08-09

**Authors:** Junseong Park, Jin-Kyoung Shim, Mirae Lee, Dokyeong Kim, Seon-Jin Yoon, Ju Hyung Moon, Eui Hyun Kim, Jeong-Yoon Park, Jong Hee Chang, Seok-Gu Kang

**Affiliations:** 1 Department of Neurosurgery, Brain Tumor Center, Severance Hospital, Yonsei University College of Medicine, Seoul, 03722 Republic of Korea; 2 Precision Medicine Research Center, College of Medicine, The Catholic University of Korea, Seoul, 06591 Republic of Korea; 3 Brain Tumor Translational Research Laboratory, Severance Biomedical Research Institute, Yonsei University College of Medicine, Seoul, 03722 Republic of Korea; 4 Department of Neurosurgery, The Spine and Spinal Cord Institute, Gangnam Severance Hospital, Yonsei University College of Medicine, Seoul, 06230 Republic of Korea; 5 Department of Biochemistry and Molecular Biology, College of Medicine, Yonsei University, Seoul, 03722 Republic of Korea; 6 Department of Biomedicine & Health Sciences, College of Medicine, The Catholic University of Korea, Seoul, 06591 Republic of Korea; 7 Department of Medical Science, Yonsei University Graduate School, Seoul, 03722 Republic of Korea

**Keywords:** CNS cancer, Targeted therapies, CNS cancer, Translational research, Molecular medicine

## Abstract

**Background:**

Glioblastoma (GBM), one of the most lethal tumors, exhibits a highly infiltrative phenotype. Here, we identified transcription factors (TFs) that collectively modulate invasion-related genes in GBM.

**Methods:**

The invasiveness of tumorspheres (TSs) were quantified using collagen-based 3D invasion assays. TF activities were quantified by enrichment analysis using GBM transcriptome, and confirmed by cell-magnified analysis of proteome imaging. Invasion-associated TFs were knocked down using siRNA or shRNA, and TSs were orthotopically implanted into mice.

**Results:**

After classifying 23 patient-derived GBM TSs into low- and high-invasion groups, we identified active TFs in each group—PCBP1 for low invasion, and STAT3 and SRF for high invasion. Knockdown of these TFs reversed the phenotype and invasion-associated-marker expression of GBM TSs. Notably, MRI revealed consistent patterns of invasiveness between TSs and the originating tumors, with an association between high invasiveness and poor prognosis. Compared to controls, mice implanted with STAT3- or SRF-downregulated GBM TSs showed reduced normal tissue infiltration and tumor growth, and prolonged survival, indicating a therapeutic response.

**Conclusions:**

Our integrative transcriptome analysis revealed three invasion-associated TFs in GBM. Based on the relationship among the transcriptional program, invasive phenotype, and prognosis, we suggest these TFs as potential targets for GBM therapy.

## Background

Glioblastoma (GBM), the most common primary brain tumor, is associated with poor prognosis and high mortality [1], despite the application of the best treatment modalities [2–4]. Although several efforts have been made to develop molecules for targeted therapy, they have failed to improve the overall survival of GBM patients [5]. To date, there are no clinically effective targeted therapies for GBM, underscoring the urgent need for new conceptual approaches to overcome treatment failure. In the process of malignant progression, GBM is characterized by an infiltrative phenotype and resistance to conventional therapies [6]. Its malignant features are related to the stem-like cells present at the invasive front [7], which have been linked to tumorspheres (TSs) isolated in vitro from GBM tissues [8]. Accordingly, TSs derived from GBM patients are considered as good model platforms for testing drug effects and characterizing specific features of GBM, including stemness and invasiveness [9–12]. Therefore, we used GBM TSs and murine orthotopic xenograft models in this study.

Migratory and invasive capabilities, together with mesenchymal transition and subsequent distant metastasis, are hallmarks of most solid tumors [13]. Similarly, invasiveness is a major challenge in the clinical management of glioma [14, 15]. We previously reported that the invasive subtype of GBM is associated with a poorer prognosis than the mitotic subtype [16]. However, no effective therapeutic interventions targeting invasion are currently available for GBM, partially owing to the diversity and redundancy of invasion-machinery genes [17–19]. Accumulating evidence suggests that current therapeutic modalities, including radiotherapy and anti-angiogenic therapy, may instead enhance GBM invasiveness [14, 20, 21]. These observations highlight the importance of identifying targets causally linked to invasion.

To address this need, we analyzed transcriptome data from GBM patient-derived primary TSs and identified transcription factors (TFs) capable of modulating invasion-related genes in GBM TSs. In addition to GBM TSs, we used mouse orthotopic xenograft models, patient clinical data, and The Cancer Genome Atlas (TCGA) datasets for robust validation. Based on our findings, we propose the identified invasion-deterministic TFs as potential targets for developing new GBM therapeutic strategies.

## Methods

### Patient information, GBM TS isolation, and 3D invasion assay

We studied 23 *IDH1* wild-type GBM patients, newly diagnosed and with no history of surgery, chemotherapy, or radiotherapy (Supplementary Table S1). MR images were captured using the Achieva 3.0 T system (Philips Medical Systems) within 7 d before removal of the respective brain tumor. Axial images were taken parallel to the anterior and posterior limbs of the corpus callosum. The invasion was quantified as the area occupied by the tumor, [T2 FLAIR − T1 contrast-enhanced (CE)]/T1 CE, as suggested in a previous study [22]. TS-forming GBM cells were established from fresh patient tissue specimens [10]. For TS culture [12, 23–25], cells were cultured in TS complete medium comprising DMEM/F-12 (Mediatech), 1× B27 (Invitrogen), 20 ng/mL bFGF, and 20 ng/mL EGF (Sigma-Aldrich). For 3D invasion assays [12], each well of a 96-well plate was filled with a mixed matrix comprising Matrigel, collagen type I (Corning Incorporated), and TS complete medium. Single spheroids were seeded inside the matrix prior to gelation, followed by the addition of TS complete medium over the gelled matrix to prevent drying. The invaded area was quantified as the occupied area after 72 h of culture relative to the occupied area at the start of culture, as (72 h – 0 h)/0 h.

### Analysis of gene expression profile

Total RNA was extracted from GBM TSs and their matched patient tissues using a Qiagen RNeasy Plus Mini kit according to the manufacturer’s protocol, and loaded onto an Illumina HumanHT-12 v4 Expression BeadChip (Illumina). After applying variance-stabilizing transformation, the data were quantile-normalized using the Bioconductor lumi package in R [26]. Using GENE-E, hierarchical clustering was performed with Pearson’s correlation as a distance metric (average linkage), and expression levels were depicted as heat maps. Each GBM sample was classified into Verhaak’s molecular subtypes [27] and prognostic subtypes [16] using previously defined gene sets. An enrichment map was constructed using Cytoscape [28] with the ClueGO [29] plug-in. The relative proportions of tumor-infiltrating leukocytes (TILs) were deconvoluted using CIBERSORT [30] and EPIC [31] algorithms. Some inferred cell types were collapsed into single terms, and those comprising <10% of the total TILs in all samples were filtered out to ensure clear visualization. For CIBERSORT, the algorithm was run using LM22 (the default signature matrix of 547 genes for 22 TIL types) as a reference gene expression set with 500 permutations. The fraction of stromal and immune cells was inferred from bulk tumor tissue samples using the default ESTIMATE R script (141 signature genes) [32]. The preprocessed TCGA GBM RNA-seq dataset and survival information of GBM patients were obtained from UCSC Xena Browser, and only *IDH1* wild-type samples were used (*n* = 147). For TF selection, single-sample gene set enrichment analysis (ssGSEA) was applied to the expression profiles of GBM TSs using TF-target gene sets retrieved from MSigDB c3.tft. Enrichment scores calculated from the same TFs were averaged to obtain a single value for each TF. The resulting scores were quantile-normalized across all samples and compared between the low- and high-invasion GBM TS groups via two-tailed Student’s *t* tests. Functional interactions among the selected TFs were constructed as a network map using Cytoscape with the Reactome FI [33] plug-in. Scripts used for the analyses presented in this study are available from the corresponding author upon request.

### Knockdown of TFs

For in vitro experiments, Lipofectamine 3000 (Invitrogen) was used to transfect dissociated GBM TSs with AccuTarget Predesigned siRNA duplexes (Bioneer) targeting human STAT3 (#6774), SRF (#6722), or PCBP1 (#5093); a random siRNA sequence was used as the negative control. Sphere formation was detected 72 h post transfection, and single spheres were used in the 3D invasion assays. For in vivo implantation, TS15-88-luc cells were transduced with ready-to-use lentiviral particles expressing shRNA (Santa Cruz Biotechnology) for STAT3 (#sc-29493-V) or SRF (#sc-36563-V). Transduction efficiency was monitored by co-transducing lentiviral particles containing copGFP gene (#sc-108084), together with targeting shRNA, at a 1:4 ratio (i.e., 20% copGFP lentiviral particles). Cells transduced with copGFP lentiviral particles only were used as control. After transduction, cells stably expressing shRNAs were isolated via puromycin selection.

### Cell-magnified analysis of the proteome (MAP) imaging

Sphere-formed TS14-15 and TS13-64 cells were seeded in 24-well plates and fixed by incubating with 4% paraformaldehyde for 15 min. The cells were then thoroughly embedded in a MAP hybrid polymer by adding 30 μL of the cell-MAP solution, as previously described [34]. The cell-MAP solution was quickly added to the coverslip and polymerized for 5 min. The gels were then peeled off the coverslip, washed thoroughly, and incubated for 30 min in clearing solution at 37 °C. The cell-MAP gels were then cut into pieces and washed thoroughly again. Cells embedded in the clarified, expanded gels were permeabilized with 0.2% Triton X-100 (Sigma-Aldrich) in 0.1 M PBS for 5 min, then blocked with 1% BSA-PBST solution for 1 h. Immunostaining was performed via incubation with antibodies against STAT3, SRF, or PCBP1 (1:500) for 3 d, followed by incubation with a secondary antibody (1:1000) for 2 d. After washing the cells with 0.1 M PBS solution for 2 h, the cells were counterstained with DAPI at room temperature for 15 min. The cell-MAP gels were incubated in distilled water until they reached 4-fold expansion (~12 h). Before imaging, the labeled gels were transferred to 35 mm confocal dishes, fixed with a small amount of distilled water, and overlaid with 25 mm round coverslips.

### Western blotting

Proteins in cell lysates were separated via SDS-PAGE using 10% Tris-glycine gels, transferred to nitrocellulose membranes, and incubated with antibodies against STAT3 (9139 S, Cell Signaling Technology); β-catenin (610154, BD Biosciences); SRF (sc-25290), GAPDH (sc-32233, Santa Cruz Biotechnology); N-cadherin (MAB13881, R&D Systems); PCBP1 (ab74793), Zeb1 (ab203829, Abcam). The proteins were detected using horseradish peroxidase-conjugated IgG (Santa Cruz Biotechnology) with SuperSignal West Femto Maximum Sensitivity Substrate and SuperSignal West Pico PLUS Chemiluminescent Substrate (Thermo Fisher Scientific). Images were captured using Amersham Imager 600 (GE Healthcare Life Sciences).

### Mouse orthotopic xenograft model and bioluminescence imaging

We used male athymic nude mice (6 wk old; Central Lab. Animal Inc.). The mice were housed in micro-isolator cages under sterile conditions and observed for at least 1 week before study initiation, to ensure proper health. Lighting, temperature, and humidity were centrally controlled. Dissociated GBM TSs (5 × 10^5^ per mouse) were implanted into the right frontal lobe of mice at a depth of 4.5 mm, using guide-screw system [35]. The mice were randomly allocated based on their body weights without blinding (*n* =  5 mice per group). If the body weight decreased by more than 15% relative to the maximum weight, mice were euthanized according to the approved protocol. For bioluminescence acquisition and analyses, mice were injected intraperitoneally with 100 μL D-luciferin (30 mg/mL; Promega) under 2.5% isoflurane anesthesia, 15 min before signal acquisition, and then observed using IVIS imaging system and Living Image v4.2 software (Caliper Life Sciences). For immunohistochemistry, sections (5 μm thick) were obtained using a microtome and transferred onto adhesive slides. Antigen retrieval and antibody attachment were performed using the Discovery XT platform (Ventana Medical Systems). Zeb1 was detected using a peroxidase/DAB staining.

## Results

### Classification of GBM TSs according to invasiveness

**Fig. 1:**
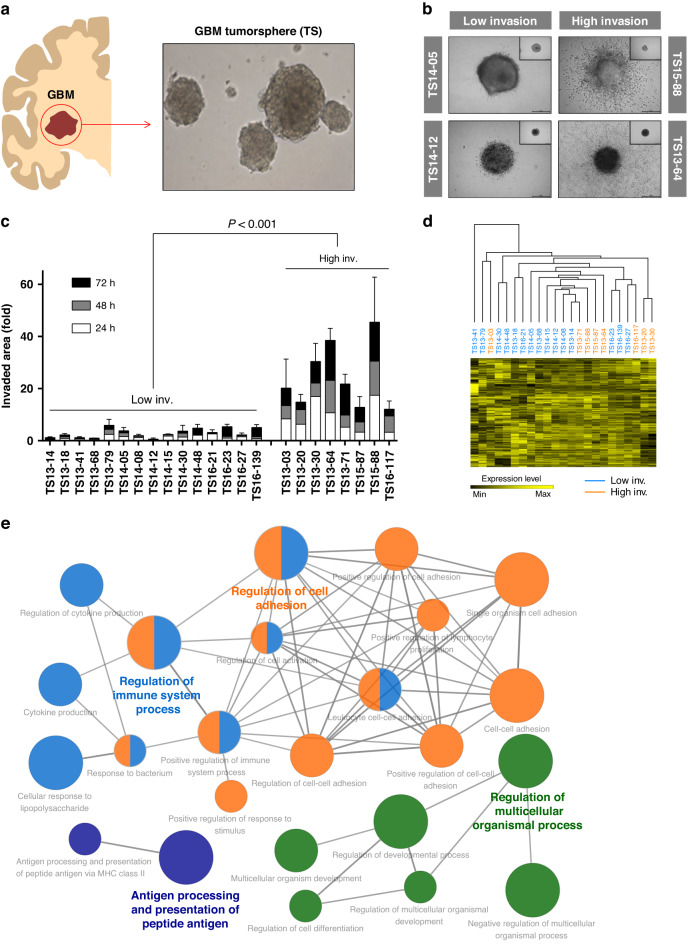
Classification of GBM TSs by invasiveness. **a** GBM patient-derived TSs were isolated and cultured. **b**,**c** TS invasiveness was evaluated using 3D invasion assay; 23 TSs were classified into low- and high-invasion groups, which were compared by two-tailed Student’s *t* test. **d** Unsupervised hierarchical clustering of TS gene expression was performed using Pearson’s correlation as the distance metric. The dendrogram shows distances among TSs. **e** Enrichment map, visualizing 402 DEGs (two-tailed Student’s *t* test; FDR < 5%) after clustering and functional annotation. Node sizes reflect the statistical significance of over-representation of GO terms (two-sided hypergeometric test; nodes with Bonferroni-adjusted *P* < 0.01 are displayed), and node color reflects module clustering: the most significant GO terms for each module are highlighted. Node edges denote the kappa score relationship.

To assess the invasiveness of GBM, we first isolated TSs from several newly diagnosed *IDH1* wild-type GBM patients (Fig. 1a). These GBM TSs have been established as a model platform to mimic the in vivo tumor microenvironment, displaying specific features of cancer stem cells [8, 10]. In total, 23 GBM TSs (Supplementary Table S1) were evaluated for invasiveness using collagen-based 3D invasion assays (Supplementary Fig. S1 and Supplementary Video S1) and classified into low- and high-invasion groups (Fig. 1b and c). General clinical parameters of GBM TS-matched patients, including age, sex, *MGMT* methylation, *EGFR* amplification, Ki-67 expression, and extent of resection (EOR), were not significantly correlated with the invasiveness of GBM TSs (Supplementary Fig. S2). In addition, unsupervised hierarchical clustering using expression levels of individual genes could not distinguish low- and high-invasion GBM TSs (Fig. 1d). Although functional annotation of differentially expressed genes (DEGs) between low- and high-invasion GBM TS groups using Gene Ontology (GO) gene sets showed the enrichment of several invasion- or cell adhesion-associated gene sets (Fig. 1e), causative molecules responsible for invasiveness could not be further specified. Therefore, we sought to identify additional deterministic targets for invasion. Given the diversity and redundancy of invasion-machinery genes [17–19], we focused on identifying transcriptional regulatory networks that can induce collective expression of invasion-related genes in GBM.

### Identification of invasion-modulating TFs in GBM

**Fig. 2:**
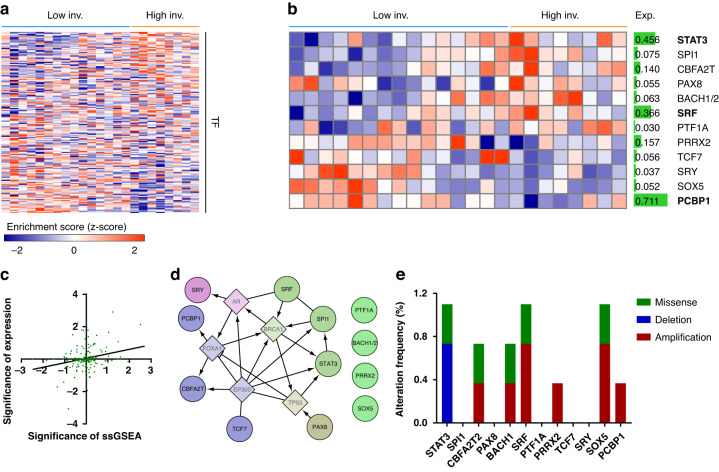
Identification of invasion-associated TFs. **a** ssGSEA was performed using curated TF-target gene sets, and enrichment scores are depicted. **b** TFs with significantly different enrichment scores (two-tailed Student’s *t*-test, FDR < 5%) between the low- and high-invasion groups are displayed. The mean expression levels of each TF are indicated using the green scale bars. **c** Scatter plot of the correlation between the ssGSEA and expression significance levels. Enrichment scores calculated in (**a**) and expression levels of each TF were compared between the low- and high-invasion groups. The significance of ssGSEA and expression is presented as −log_10_(*P*). Each dot denotes a single TF, and the black line indicates the regression line (Pearson’s correlation; *P* < 0.001, *R* = 0.26). **d** Functional interaction network for the selected TFs. Circle nodes labeled in black: TFs; diamond nodes labeled in gray: linker genes. Nodes within the same module are shown in the same color. **e** Mutation profiles of selected TFs, retrieved from TCGA GBM datasets (*n* = 645).

To quantify the contribution of each transcriptional program to GBM invasiveness, we performed ssGSEA for classified GBM TSs using curated TF-target gene sets (Fig. 2a). TFs with a significantly elevated enrichment score in the high-invasion GBM TS group, namely STAT3, SPI1, SBFA2T, PAX8, BACH1/2, SRF, and PTF1A, were predicted as invasion-promoting TFs. In contrast, TFs with a significantly elevated enrichment score in the low-invasion GBM TS group, namely PRRX2, TCF7, SRY, SOX5, and PCBP1 were predicted as invasion-suppressing TFs (*Methods* and Fig. 2b). The significance of these ssGSEA scores positively correlated with the significance of expression levels of the corresponding TFs, validating this method and the TF-target gene relationship in GBM TSs (Fig. 2c). The functional interaction network among selected TFs is presented in Fig. 2d. Mutation frequencies of these TFs were not high in TCGA GBM dataset, implying that other factors, including transcriptional regulation, altered the TF activities in each GBM TS (Fig. 2e). Among these TFs, those with high mean expression levels in GBM TSs were finally selected as potential invasion-modulating TFs: STAT3, SRF, and PCBP1 (Fig. 2b).

**Fig. 3:**
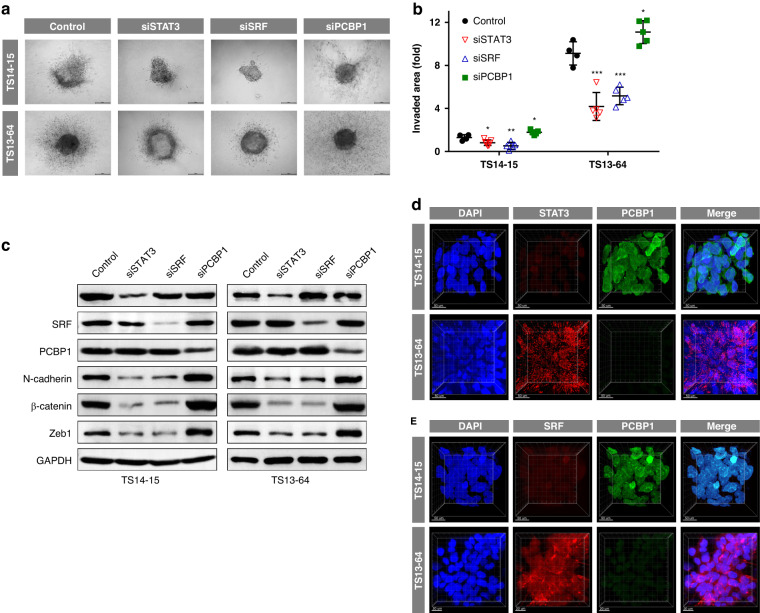
Validation of invasion-associated TFs. TS14-15 and TS13-64 cells were transfected with siRNA for STAT3, SRF or PCBP1, and 3D invasion assays and western blotting were performed 72 h post-transfection. **a**,**b** Invaded areas were evaluated as the increase in occupied area at 72 h relative to the area at 0 h, normalized to the initial occupied area [(72 h–0 h)/0 h]. Groups were compared using one-way ANOVA with Tukey’s post hoc test (*P < 0.05, **P < 0.01, ***P < 0.001). **c** Knockdown efficiency of siRNAs targeting STAT3, SRF, and PCBP1, and expression of invasion-associated genes were assessed via western blotting. **d**,**e** Expression of STAT3, SRF (red), and PCBP1 (green) were evaluated via cell-MAP imaging. DAPI (blue) was used to counterstain the nuclei.

To validate whether the predicted TFs modulate invasiveness in GBM TSs, we downregulated the expression of each TF in the low- (TS14-15) and high- (TS13-64) invasion groups using siRNA. At 72 h post-transfection of siRNA against STAT3, SRF, or PCBP1, the GBM TSs in both groups were assessed via in vitro 3D invasion assay. Knockdown of STAT3 or SRF, which were identified as invasion-promoting TFs, significantly reduced the invasiveness of these GBM TSs. In contrast, knockdown of PCBP1, which was identified as an invasion-suppressing TF, increased it significantly (Fig. 3a and b). Western blot analyses confirmed the molecular basis of these results. Knockdown of STAT3 or SRF reduced the expression of invasion- and mesenchymal transition-associated proteins (N-cadherin, β-catenin, and Zeb1), whereas knockdown of PCBP1 increased them (Fig. 3c and Supplementary Fig. S3). We also evaluated the expression of STAT3, SRF, and PCBP1 in GBM TSs via cell-MAP imaging. TS14-15 (low-invasion GBM TS) showed higher expression of PCBP1 than TS13-64 (high-invasion GBM TS), whereas TS13-64 showed higher expression of STAT3 and SRF than TS14-15 (Fig. 3d and e and Supplementary Video S2). These findings suggest the following invasion-deterministic TFs: PCBP1 for low invasion, and STAT3 and SRF for high invasion.

### Consistent invasiveness in GBM patients and paired TSs

**Fig. 4:**
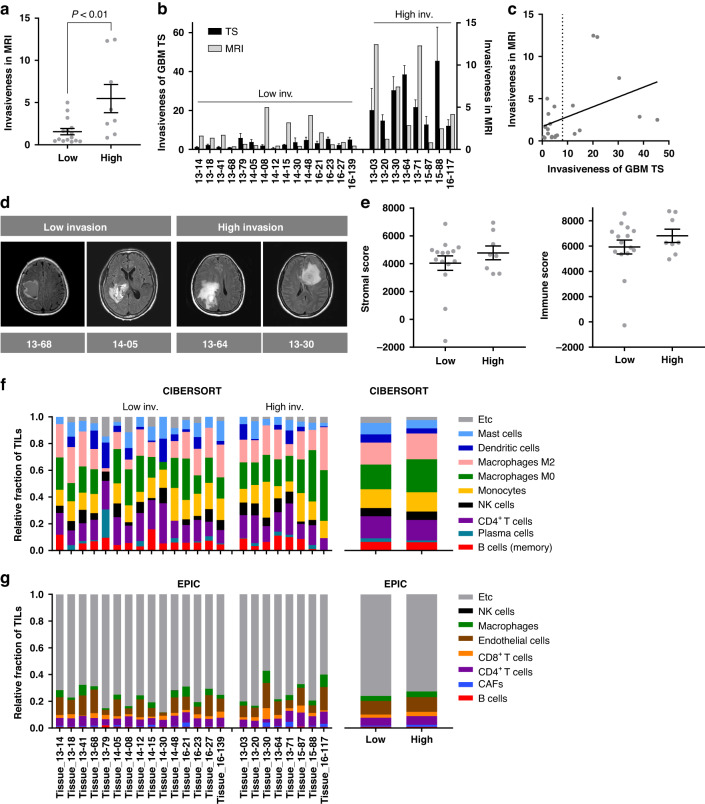
Tumor invasiveness in TS-matched GBM patients. **a**–**d** Tumor invasiveness, evaluated via MRI, was compared between the low- and high-invasion TS-paired GBM patients using two-tailed Student’s *t* test (**a**). Invaded area of paired samples from TSs (via 3D invasion assay) and GBM patient tumors (via MRI) shown as bar graph (**b**) and scatter plot (Spearman’s correlation; *P* < 0.05, *R* = 0.42) (**c**). Representative images are displayed in (**d**). **e** Stromal and immune scores of GBM tissues, calculated using ESTIMATE. **f**,**g** Relative fraction of TIL subsets in GBM tissues, estimated using CIBERSORT (**f**) and EPIC (**g**). In (**e**–**g**), there were no significant differences between the low- and high-invasion TS groups (two-tailed Student’s *t* test).

Next, we evaluated invasiveness in TS-matched GBM patients using MRI and found patterns consistent with TS invasion, suggesting that GBM TSs are good models that reasonably reflect the invasiveness of tumors from matched GBM patients (Fig. 4a–d). Owing to this, the therapeutic targets identified by in vitro 3D assays of GBM TSs could be applied in a clinical setting. To determine whether differences in invasiveness arose from intrinsic or extrinsic factors of tumor cells, we computationally inferred the stromal composition in bulk tissues from TS-matched GBM patients. Stromal and immune scores, calculated via the ESTIMATE method, did not differ significantly between the low- and high-invasion GBM TS groups (Fig. 4e). In addition, the relative proportion of TILs, estimated using the CIBERSORT and EPIC algorithms, showed similar patterns between these groups (Fig. 4f and g), suggesting that differential invasiveness of GBM was not originated from the tumor stromal microenvironment, but instead caused by intrinsic factors of tumor cells.

### Relationship between invasiveness and prognosis in GBM

**Fig. 5:**
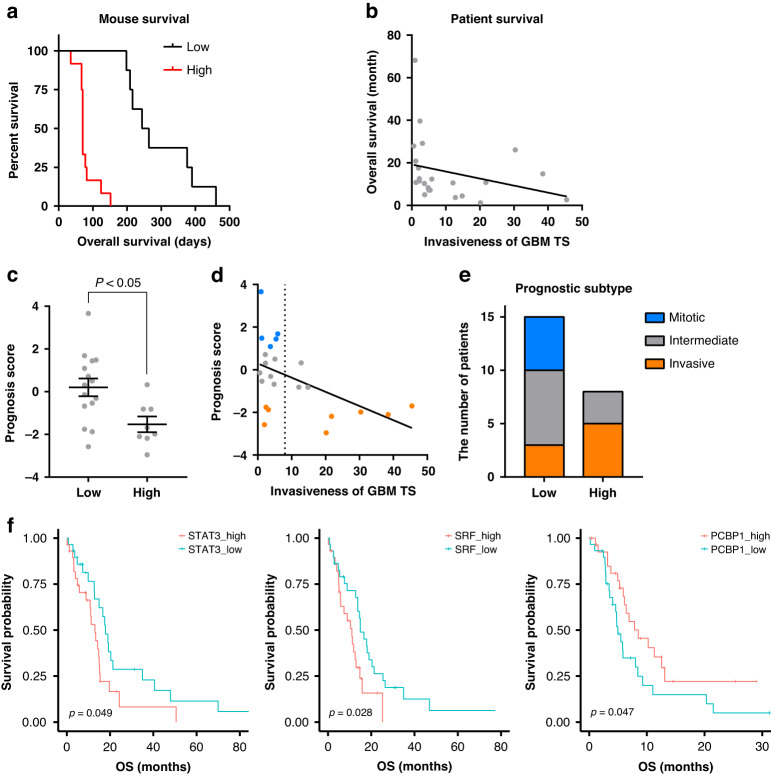
Relationship between invasiveness and prognosis. **a** Low- or high-invasion TSs were implanted in mouse brains, and survival probability was estimated using Kaplan–Meier curves (log-rank test; *P* < 0.001). **b** Scatter plot of TS invasiveness and the overall survival of paired GBM patients (Spearman’s correlation; *P* < 0.01, *R* = − 0.55). **c** Prognosis scores of TS-paired GBM patients, compared by two-tailed Student’s *t* test. **d** Scatter plot of TS invasiveness and prognosis scores of paired GBM patients (Spearman’s correlation; *P* < 0.05, *R* = –0.46). **e** Prognostic subtype of TS-paired GBM patients (Fisher’s exact test; *P* < 0.05). **f** TCGA GBM RNA-seq dataset (*IDH1* wild-type; *n* = 147) was divided into low- and high-enrichment score groups (selecting the lowest and highest 20% of samples), calculated using target gene sets of invasion-deterministic TFs (Fig. 2). Survival probability was estimated based on Kaplan–Meier curves, and statistical significance was determined using the log-rank test.

We further investigated the relationship between invasiveness and prognosis in GBM. After implanting low- or high-invasion GBM TSs in mouse brains, we estimated survival probability via Kaplan–Meier curves (Fig. 5a). The overall survival of several TS-matched GBM patients was evaluated using the same method (Fig. 5b). In both cases, the low-invasion group showed a significantly prolonged survival time, indicating a relationship between invasiveness and prognosis in GBM. Consistent with this, the invasiveness of GBM TSs was correlated with prognostic subtypes of GBM, previously identified by our group [16], despite the absence of significant enrichment of Verhaak’s molecular subtype according to invasiveness (Supplementary Fig. S4). Prognosis scores were significantly reduced in GBM patients paired with high-invasion TSs (Fig. 5c and d). Moreover, the mitotic subtype was only observed in the low-invasion group, whereas the invasive subtype was enriched in the high-invasion group (Fig. 5e). To confirm the involvement of transcriptional regulatory networks in this association between invasiveness and prognosis, we assessed three invasion-deterministic TFs in TCGA GBM datasets. TCGA samples were divided into low- and high-groups according to the enrichment scores of these TFs, calculated via the same method used in Fig. 2, and the overall survival was compared using Kaplan–Meier curves. For invasion-promoting TFs (STAT3 and SRF), the low-enrichment score group showed significantly longer overall survival, whereas the invasion-suppressing TF (PCBP1) showed the opposite pattern (Fig. 5f). These data collectively suggest that invasiveness is inversely correlated with prognosis in GBM, as with many other types of solid tumors, indicating a possible therapeutic strategy targeting invasiveness.

### Efficacy of invasion-targeted therapy in a mouse orthotopic xenograft model

**Fig. 6:**
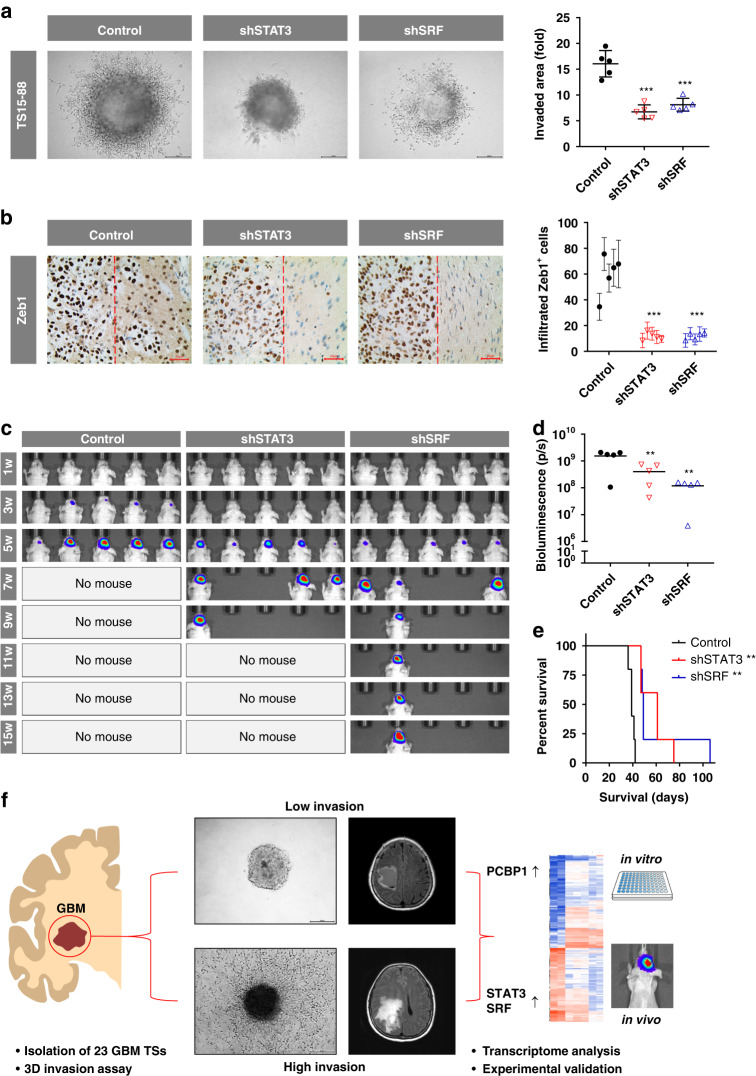
Efficacy of invasion-targeted therapy in a mouse orthotopic xenograft model. In vivo therapeutic responses to invasion targeting were assessed by constructing TSs (TS15-88) stably expressing shRNA against STAT3 or SRF, and evaluating their phenotypes in a mouse orthotopic xenograft model (*n* =  5 mice per group). **a** TS invasiveness was evaluated using 3D invasion assay (one-way ANOVA with Tukey’s post hoc test for multiple comparisons; ***P < 0.001). **b** Brain sections obtained from euthanized mice were immunostained for Zeb1 (brown) and counterstained with hematoxylin (blue) to probe invading tumor cells (left). The number of infiltrated Zeb1^+^ cells (right of the red dashed line) was determined from 10 images for each mouse and compared among the group (mean ± SEM; two-way ANOVA with Tukey’s post hoc test; ***P < 0.001 compared with controls). **c**,**d** Tumor volume was measured using bioluminescence imaging. Signal intensity was quantified as the sum of all detected photons (total flux; one-way ANOVA with Tukey’s post hoc test; **P < 0.01). **e** Kaplan–Meier curves were used to estimate survival probability for each group (log-rank test with Bonferroni’s post hoc test; **P < 0.01). **f** Summary of the findings.

To evaluate in vivo therapeutic responses toward targeting invasiveness, we constructed cell lines from high-invasion GBM TS (TS15-88) stably expressing shRNA against invasion-promoting TFs. Transduction of lentiviral particles and knockdown efficiencies of STAT3 or SRF were validated using fluorescence imaging (Supplementary Fig. S5). These GBM TSs showed significantly reduced invasiveness in 3D invasion assays (Fig. 6a), similar to the results following siRNA-mediated knockdown (Fig. 3). To quantify in vivo invasiveness via immunohistochemistry, we assayed for Zeb1 expression in the brain tissue obtained from orthotopic xenograft model mice sacrificed at the same time (4 wk after inoculation with shRNA-transduced TS15-88). The number of invading cells, indicated by Zeb1^+^ cells infiltrating outside the gross tumor mass, was significantly reduced by STAT3 or SRF knockdown. In addition, staining images showed that the tumor margins were smoother in the STAT3- and SRF-knockdown groups (Fig. 6b). Bioluminescence imaging revealed that tumor growth was significantly lower in both STAT3- and SRF-knockdown groups than in the control (Fig. 6c and d). These groups also exhibited significantly prolonged survival (Fig. 6e). Collectively, these observations demonstrate the in vivo anticancer efficacy of invasion-targeted therapy using STAT3- or SRF-knockdown. The summarized results of this study are presented in Fig. 6f.

## Discussion

Intertumoral heterogeneity and distinct molecular subtypes among patients are well-known phenomena in solid tumors [36], including GBM, which has several subtypes according to its molecular features [27] or prognosis [16]. Similarly, we have previously shown that GBM TSs exhibit heterogeneity in terms of morphology, molecular subtype, and drug sensitivity [12, 15, 23, 24]. The current findings indicate that GBS TSs can be clearly divided into two distinct biological phenotypes based on invasiveness. Thus, despite intertumoral heterogeneity among GBM TSs, invasiveness can be a robust classifier of GBM, reflecting its biological phenotype, prognosis, and molecular signature.

The effects of blocking single target genes are often counterbalanced by the homeostatic expression of other genes with similar functions, which frequently bypasses or mitigates the efficacy of targeted therapy [37]. As single TFs regulate the expression of multiple target genes with similar functions [38, 39], blocking one TF can exert effects similar to inhibiting multiple downstream target genes. Therefore, utilizing TFs as drug targets can help to overcome these limitations [40, 41]. STAT3 is linked to tumor progression in a wide variety of cancers, and its expression promotes invasion and metastasis in ovarian [42] and breast cancer [43]. SRF also plays an important role in tumor progression, facilitating invasion and metastasis in gastric cancer [44, 45].

Tumor-stroma interactions have been extensively investigated, and several stromal factors are known to promote tumor cell invasion or metastasis [46]. For instance, in GBM, tumor mesenchymal stem-like cell-mediated expression of C5α augments the invasiveness of GBM TSs [47, 48]. In contrast, the current findings reveal invasion-deterministic factors affiliated specifically with tumors: GBM TSs closely mirrored the invasiveness of the tumors from which they were derived, thus making it unnecessary to consider patient-derived stromal cells. Moreover, our transcriptome analysis of GBM tissues showed that the differences in invasiveness originated from tumor cells, not from infiltrating stromal cells, suggesting potential therapeutic opportunities for targeting invasion.

An invasion-targeted strategy for GBM offers several advantages. Despite the enormous efforts devoted to developing targeted therapies for GBM, no chemical agents other than temozolomide, a cytotoxic drug with several side effects, are available for GBM patients. Because invasion into normal tissue is a major hallmark of cancer not exhibited by normal cells [13], drugs that target invasiveness can enhance cancer specificity. Moreover, despite many reports demonstrating positive correlations between EOR and prognosis [4, 49], prudence is warranted in increasing the EOR of brain tumors, owing to potential impacts on the central nervous system and associated loss of cognitive function. The need to limit EOR contributes to frequent post-surgical relapse of GBM. The subventricular zone, which is distinct from the tumor region, has recently been proposed as the origin of GBM [50, 51]. Therefore, re-invasion of cancer cells from this region could be another mechanism of GBM recurrence after therapy, a possibility that increases the need for new therapeutic strategies. Since infiltration of tumor cells into normal tissue obscures the margin of the tumor area, targeting invasiveness as a new adjuvant therapy could maximize the effects of surgical resection and reduce the probability of recurrence. In addition, invasion-targeted therapy could be combined with other therapeutic modalities, including temozolomide and radiotherapy, while avoiding mechanistic overlap.

By analyzing the transcriptome data of GBM patient-derived primary TSs, we identified STAT3, SRF, and PCBP1 as deterministic TFs capable of inducing the collective expression of invasion-associated genes in GBM. Consistent with our previous findings [16], highly invasive GBM TSs were associated with worse prognosis in GBM patients and mice. Using siRNA- and shRNA-mediated knockdown of the pro-invasion TFs STAT3 and SRF, we evaluated the efficacy of invasion-targeted therapy in GBM, identifying these TFs as potential drug targets for new therapeutic strategies against GBM (Fig. 6f).

### Supplementary information

Supplementary Material

Supplementary Video S1

Supplementary Video S2A

Supplementary Video S2B

Supplementary Video S2C

Supplementary Video S2D

ARRIVE guidelines 2.0

## Data Availability

Microarray datasets are available in the Gene Expression Omnibus repository: GSE159000 (GBM TSs) and GSE131837 (GBM tissues).
