# Supplementary material for: Classification of IDH wild-type glioblastoma tumorspheres into low- and high-invasion groups based on their transcriptional program
Source: Br J Cancer. 2023 Aug 9;129(7):1061–70. doi: 10.1038/s41416-023-02391-y (PMC10539507; doi:10.1038/s41416-023-02391-y)
Supplement: Supplementary file 1 — Supplementary Material [file 41416_2023_2391_MOESM1_ESM.pdf]

Classification of IDH Wild-Type Glioblastoma Tumorspheres into Low- and High-Invasion Groups according to the Transcriptional Program

Junseong Park, Jin-Kyoung Shim, Mirae Lee, Dokyeong Kim, Seon-Jin Yoon, Ju Hyung Moon, Eui Hyun Kim, Jeong-Yoon Park, Jong Hee Chang, Seok-Gu Kang

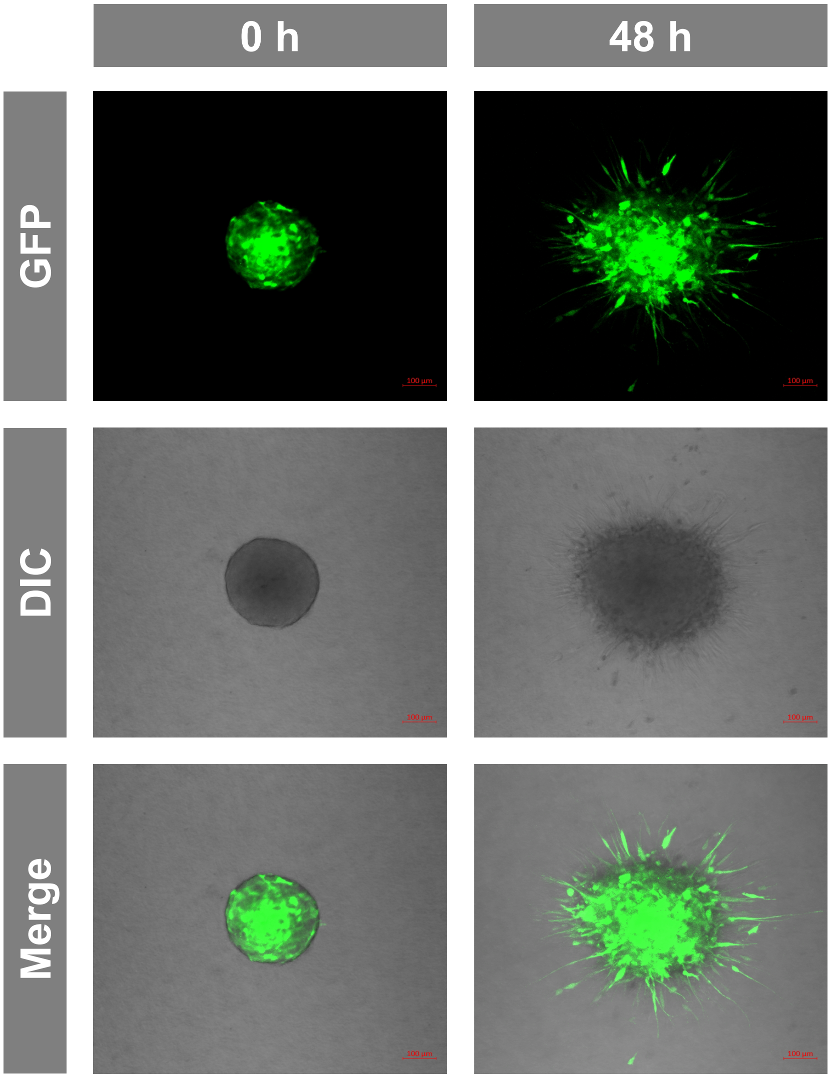

Supplementary Fig. S1. Representative images for invasion of GBM TSs.

Invasiveness of copGFP-tagged TS15-88 cells were evaluated using 3D invasion assays (see **Supplementary Video S1**).

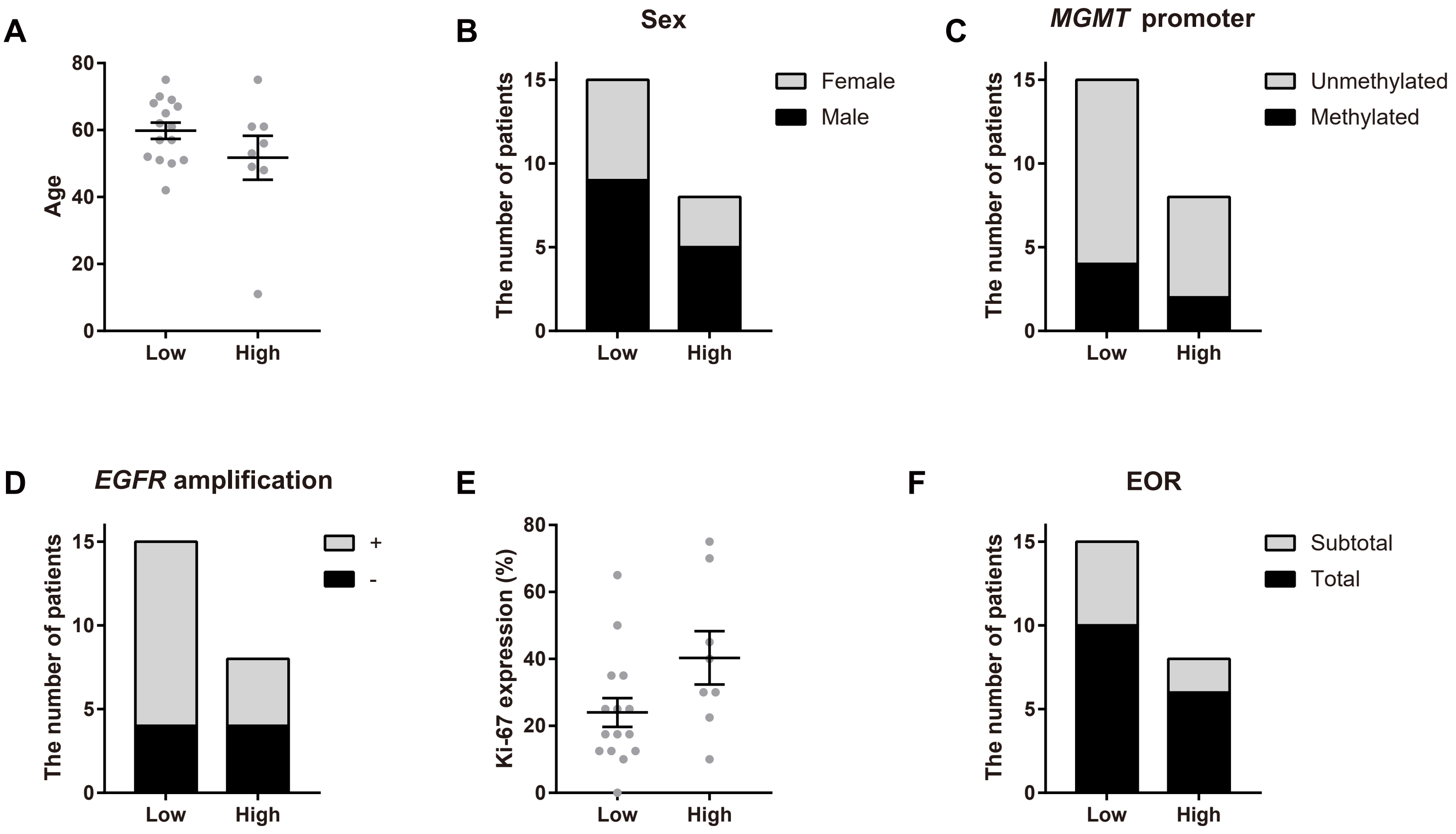

Supplementary Fig. S2. Clinical parameters of TS-matched GBM patients.

Clinical information, including age (**A**), sex (**B**), methylation status of *MGMT* promoter (**C**), *EGFR* amplification (**D**), Ki-67 expression (**E**), and EOR (**F**) were evaluated in TS-matched GBM patients. There were no significant differences between low- and high-invasion GBM TS groups (for (**A**) and (**E**), two-tailed Student's *t*-test; for (**B**), (**C**), (**D**), and (**F**), Fisher's exact test).

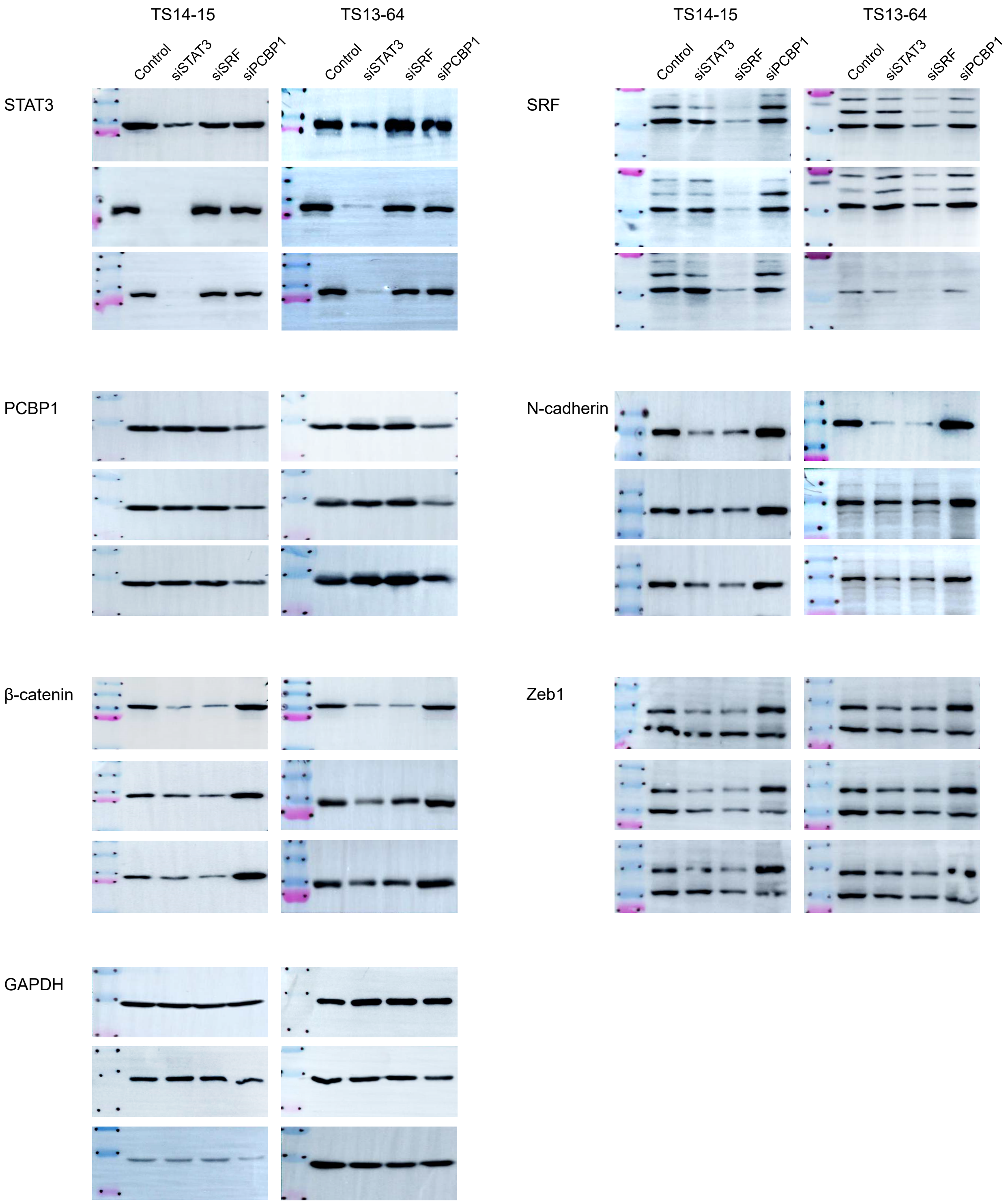

Supplementary Fig. S3. Raw data of western blot.

Raw data of western blot in **Fig. 3C** were presented including protein ladders. All experiments were performed at least three times.

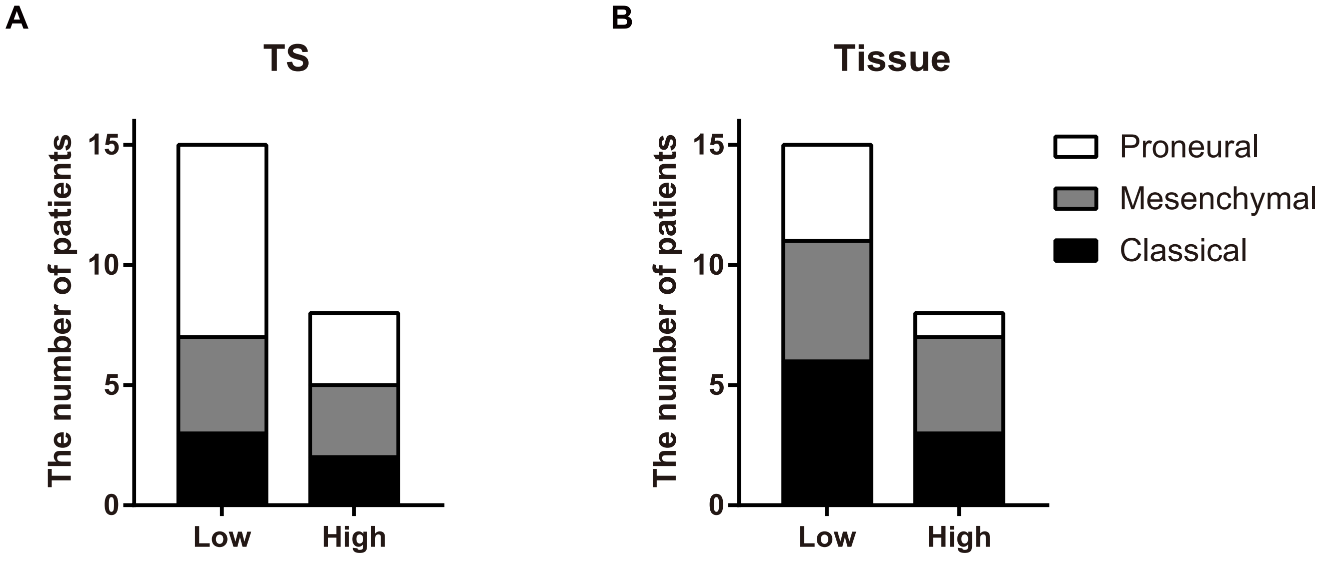

Supplementary Fig. S4. Verhaak's molecular subtype.

Expression profiles of GBM TSs (**A**) and tissues obtained from their matched patients (**B**) were assorted by Verhaak's molecular subtypes. The proportion of subtypes were not significantly different between low- and high-invasion GBM TS groups by Fisher's exact test.

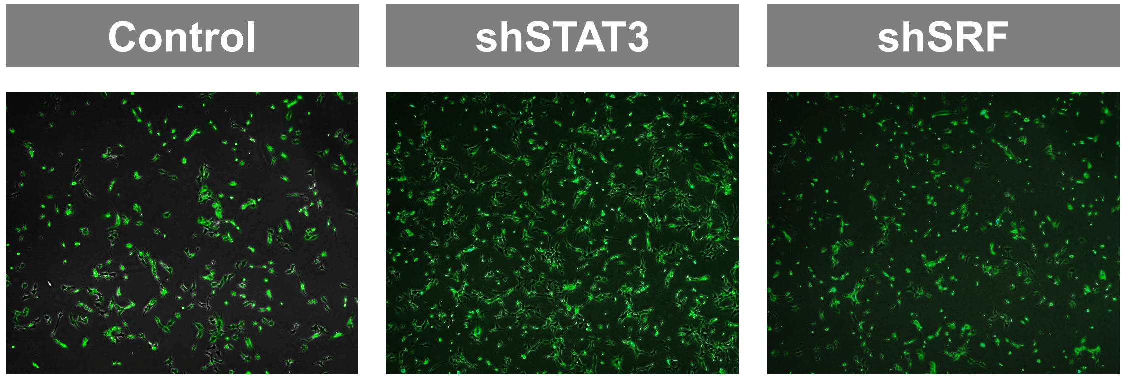

Supplementary Fig. S5. Transduction of lentiviral particles.

Transduction efficiencies of lentiviral particles were monitored by fluorescence imaging of copGFP gene.

Supplementary Table S1. Clinical characteristics of the samples in this study.

Feature	Low inv. (n = 15)	High inv. (n = 8)
Age, years (median)	61	54.5
Sex (M/F)	9/6	5/3
IDH1 mutation	0	0
1p19q co-deletion	0	0
MGMT methylation in promoter	4	2
EGFR amplification	11	4
Ki-67 expression (median)	17.5	35
Prognostic subtype (invasive/intermediate/mitotic)	3/7/5	5/3/0
Verhaak's subtype_patient tissue (classical/mesenchymal/proneural)	6/5/4	3/4/1
Verhaak's subtype_TS (classical/mesenchymal/proneural)	3/4/8	2/3/3

Supplementary Video legends

Supplementary Video S1. Representative video for invasion of GBM TSs.

Invasiveness of copGFP-tagged TS15-88 cells were evaluated using 3D invasion assays (see **Supplementary Fig. S1**).

Supplementary Video S2. Cell-MAP imaging of GBM TSs.

Expression levels of STAT3, SRF (red), and PCBP1 (green) were evaluated by cell-MAP imaging. DAPI (blue) was used to counterstain nuclei. (**A**) TS14-15 stained for STAT3 and PCBP1. (**B**) TS14-15 stained for SRF. (**C**) TS13-64 stained for STAT3 and PCBP1. (**D**) TS13-64 stained for SRF.
